# Content and quality of websites supporting self-management of chronic breathlessness in advanced illness: a systematic review

**DOI:** 10.1038/npjpcrm.2016.25

**Published:** 2016-05-26

**Authors:** Tim Luckett, Rebecca Disler, Annmarie Hosie, Miriam Johnson, Patricia Davidson, David Currow, Anthony Sumah, Jane Phillips

**Affiliations:** 1 Faculty of Health, University of Technology Sydney (UTS), Sydney, NSW, Australia; 2 Hull York Medical School, University of Hull, Hull, East Yorkshire, UK; 3 School of Nursing, Johns Hopkins University, Baltimore, MD, USA; 4 Palliative and Supportive Services, Flinders University, Adelaide, SA, Australia

## Abstract

Chronic breathlessness is a common, burdensome and distressing symptom in many advanced chronic illnesses. Self-management strategies are essential to optimise treatment, daily functioning and emotional coping. People with chronic illness commonly search the internet for advice on self-management. A review was undertaken in June 2015 to describe the content and quality of online advice on breathlessness self-management, to highlight under-served areas and to identify any unsafe content. Google was searched from Sydney, Australia, using the five most common search terms for breathlessness identified by Google Trends. We also hand-searched the websites of national associations. Websites were included if they were freely available in English and provided practical advice on self-management. Website quality was assessed using the American Medical Association Benchmarks. Readability was assessed using the Flesch–Kincaid grades, with grade 8 considered the maximum acceptable for enabling access. Ninety-one web pages from 44 websites met the inclusion criteria, including 14 national association websites not returned by Google searches. Most websites were generated in the USA (*n*=28, 64%) and focused on breathing techniques (*n*=38, 86%) and chronic obstructive pulmonary disease (*n*=27, 61%). No websites were found to offer unsafe advice. Adherence to quality benchmarks ranged from 9% for disclosure to 77% for currency. Fifteen (54%) of 28 written websites required grade ⩾9 reading level. Future development should focus on advice and tools to support goal setting, problem solving and monitoring of breathlessness. National associations are encouraged to improve website visibility and comply with standards for quality and readability.

## Introduction

Chronic breathlessness is a common, burdensome and distressing symptom in people with advanced chronic illness, including chronic obstructive pulmonary disease (COPD), heart failure, cancer (especially lung) and neurological diseases.^1^ The experience of breathlessness is individual and multidimensional, involving ‘sensory-perceptual’, ‘affective’ and ‘impact’ components.^2^ Breathlessness may have a range of interacting causes,^3^ as well as complex relationships with other symptoms, notably anxiety and fatigue.^4^ It is an independent predictor of mortality^5^ and associated with negative impacts on activities of daily living and quality of life.^6^ Many people with chronic breathlessness periodically experience acute-on-chronic symptoms, sometimes termed breathlessness crises,^7^ which often lead to emergency presentations.^8–10^

Management of chronic breathlessness focuses on optimising treatment for underlying pathologies, managing reversible causes, reducing the frequency and severity of crises and minimising adverse impacts on quality of life.^3^ Self-management is essential for implementing pharmacological and non-pharmacological treatments and sustaining everyday functioning and emotional coping.^11^ Self-management interventions have been defined as those ‘focused on increasing a patient’s knowledge and understanding of actions they can take to manage breathlessness, including education plus strategies to improve coping skills or reduce anxiety or depression, cognitive behavioural/behavioural or psycho-educational interventions; psychosocial interventions; problem-solving interventions with or without goal setting and action plans; stress management; symptom coaching and breathing techniques’^12^ (pp 3–4).

Ideally, self-management involves patients working in collaboration with health professionals to ensure that strategies are evidence-based and optimally implemented.^13^ However, with the advent of the World Wide Web, patients now often search online for resources to support self-management, especially if they have limited access to services.^14^ Internet-based health information has the potential to influence patient behaviour and relations with health professionals in both positive and negative ways,^15^ giving rise to substantial research aimed at appraising and improving quality.^16,17^ However, no research to date has reviewed the quality of websites for self-management of chronic breathlessness. The current study set out to address this gap with the aims of (1) describing the content and quality of websites concerned with self-management of chronic breathlessness; (2) highlighting under-served areas to inform future content development; and (3) identifying any websites posing safety concerns.

## Results

### Inclusion

One hundred and sixty seven web pages returned by Google searches were reviewed against inclusion criteria (see Table 1), leading to inclusion of 53 web pages.

Sixteen videos were included from YouTube searches, and another 22 web pages from the websites of the Asthma Foundation New Zealand (*n*=1), American Lung Association (*n*=3), American Thoracic Society (ATS; *n*=3), Chest, Heart and Stroke Scotland (*n*=9), Canadian Lung Association (*n*=4) and Canadian Thoracic Society (*n*=2) that were not identified by Google or YouTube searches. This resulted in overall inclusion of 91 web pages from 44 websites. Table 2 summarises characteristics relating to country of origin, authorship, format, target audiences and focal health conditions across these 44 websites.

### Quality appraisal

When reviewed against the American Medical Association (AMA) Benchmarks,^18^ 24 (54%) web pages were found to give no information on authorship, 34 (77%) failed to attribute the advice they offered and 10 (23%) gave no dates of authorship of review. Thirty-nine (89%) websites provided only limited information on disclosure, and 4 (9%) offered no information at all. Among 28 websites with written content, reading grade ranged from 5 to 16 (median grade 9), with 15 (54%) failing to meet recommendations to keep reading level at grade ⩽8.^19^

### Synthesis

When coded for content using the taxonomy of Howell *et al*.^12^ 3 (7%) websites were found to include content on structured education, 38 (86%) websites included breathing techniques, 11 (25%) included cognitive symptom management, 15 (34%) included action planning, 1 (2%) website included problem solving, 23 (52%) included the use of support services and 10 (23%) websites included partnership with healthcare providers; 17 (39%) websites were considered to contain a strong self-efficacy component. No websites offered advice on goal setting. Coding content against ATS guidance^7^ identified that 17 (39%) websites provided advice on managing breathlessness crises, 3 (7%) on monitoring of breathlessness and 20 (45%) on using pharmacological and/or non-pharmacological treatments. Additional content not included in either taxonomy referred to lifestyle factors (e.g., diet, physical activity) (*n*=27, 61%), environmental factors (e.g., pollen, pollution, temperature) (19, 43%) and end-of-life considerations (3, 7%). Supplementary Table S1 provides a breakdown of the content and focal health condition(s) of each individual website.

No websites were identified as offering unsafe advice. A consumer-developed YouTube video on using common dietary ingredients to manage breathlessness that lacked evidence for effectiveness was removed from the internet shortly after being identified.

## Discussion

Breathlessness self-management websites identified by this review were most commonly generated in the USA and focused predominantly on COPD and breathing techniques. No websites were found that offered support for goal setting, suggesting that this may be an under-served area, despite acknowledgement that this is an essential component of self-management across chronic health conditions.^13^

The authors of websites rarely explained their purposes explicitly, and the majority allocated self-management limited word-space amidst more dominant content on health conditions, diagnosis and treatment. This resulted in many websites being framed from a disease-focused biomedical perspective rather than a person-centred perspective focused on ameliorating the impacts of breathlessness on daily life. Only one website included interactive features enabling content to be tailored to consumer needs and problem solving,^20^ highlighting a further area in need of content development. In the absence of goal-setting content, advice on action planning was limited to management of breathlessness crises, preventing exacerbations, going home from hospital and preparing to go on holiday. Advice on breathlessness monitoring occurred rarely, despite this being essential for assessing whether goals have been met and recognising changes in the severity and quality of breathlessness over time to inform decisions about when to seek medical attention.^7^ Website advice on when to go to Emergency typically recommended going immediately rather than suggesting incremental strategies of the kind recommended by the ATS. The need to better support people in self-managing crises is highlighted by recent research, suggesting that up to half of visits to Emergency Departments due to breathlessness may be potentially avoidable.^21,22^

A further direction for content development is identified by our finding that few websites contained information targeted at carers, despite this group’s unmet needs for information and support.^23^ Website content for carers was concerned with understanding the carer role, as well as self-care and support networks for managing physical and emotional burden. Most practical advice to carers and patients on using treatments was focused on home oxygen, with medication-related content usually limited to mechanisms of action and adverse effects. There was little information about adjusting to increasing breathlessness and frailty and deterioration in function, advance care planning and associated changes in the carer role. Few websites broached the subject of increasing breathlessness towards the end-of-life and related care needs.^24^

Compliance with AMA benchmarks for quality was found to be disappointingly low despite, long-standing attempts to promote standards of online health information.^25^ Our finding that more than half of the websites with written content were above reading grade 8 is of special concern given that COPD and chronic heart failure are associated with cognitive impairment^26^ and people may seek information at times of crisis when ease of understanding is paramount. National associations are encouraged to improve accessibility of their website content with advice and review by consumers.

Limitations of the current review concern the fact that it is unlikely to offer an exhaustive account of all relevant websites and is focused on a specified time point, which quickly becomes outdated. However, the fact that we used the most widely used search engine and terms instils confidence that we identified websites accessed by the majority of consumers. It is therefore of concern that searches did not identify many of the websites developed by national associations or authorised clinical service websites. On the other hand, given the poor visibility of authoritative websites, it is encouraging that no websites were found that offered unsafe advice or sought to gain financially by means of inaccurate information, as has been found for other health-related information.^27^

## Conclusions

Websites supporting self-management of breathlessness identified by this review were predominantly generated from the USA and focused on breathing techniques and COPD. More content is needed to support goal setting, action planning, problem solving and monitoring of breathlessness, as these are integral components of self-management in chronic illness. More websites are also needed that deal with worsening breathlessness and decline in function as illness progresses towards end-of-life and the associated information and support needs of carers. Interactive tools that can be tailored to consumer needs are especially encouraged. National associations are recommended to improve the visibility of their resources, as well as comply with standards for quality and readability of internet health information.

## Materials and methods

A review of websites for breathlessness self-management was conducted in June 2015 from Sydney, Australia.

### Search and selection

Search methods were intended to identify websites being accessed by the majority of English-speaking consumers. We searched the most widely used search engine, Google, which at the time accounted for 93% of mobile/tablet searches and 68% of desktop searches (with a further 11% being conducted via the Chinese-language engine Baidu).^28^ Because we expected self-management websites to include content in video format, we also searched www.YouTube.com, which enables any internet user to upload content directly and also houses much of the video content accessible from other websites.

Google and YouTube were searched using terms identified by Google Trends^29^ as the five most common search terms for breathlessness: ‘breathless’, ‘shortness of breath’, ‘dyspnoea’, ‘wheeze’ and ‘difficulty breathing’. We also searched websites belonging to respiratory-related national associations in English-speaking members of the Organisation for Economic Co-operation and Development. These included websites of the American Association for Respiratory Care, American Lung Association, ATS, Australian Lung Foundation, British Lung Foundation, British Thoracic Society, Canadian Lung Association, Canadian Thoracic Society, Chest, Heart and Stroke Scotland, Irish Lung Foundation and Thoracic Society of Australia and New Zealand. In addition, we searched the website of the Asthma Foundation New Zealand, because we knew that it contained advice on chronic breathlessness from a range of causes, as well as asthma.

Google results consist of individual web pages rather than websites, which are defined as connected groups of web pages regarded as a single entity and maintained by a single person or organisation.^30^ Each web page returned by the search was opened and appraised against eligibility criteria until 10 consecutive web pages failed to yield any new web pages to include. Links from web pages were followed wherever they looked likely to be relevant for self-managing breathlessness. YouTube was harder to search systematically because results are displayed in a scrolling list with related videos appearing in the right-hand margin. To most closely approximate to a consumer’s search style, we searched YouTube using each term for 1 h, clicking on video links wherever these seemed most relevant to self-management and pursuing further links as appropriate rather than returning to the original list.

### Eligibility criteria

To be included, websites needed to be freely available in English and offer support for self-management of chronic breathlessness. Websites had to go beyond information about breathlessness and its causes or medical treatments to provide practical advice, tools or demonstrations that could be used to support self-management by people with breathlessness and/or their carers. Provided content met these criteria, we also included websites designed for healthcare professionals on the basis that consumers may access these as well. Websites that referred to breathlessness without specifying acute or chronic were included, but websites that referred exclusively to asthma ‘acute’ breathlessness outside the context of chronic breathlessness were not.

### Data extraction

Using an electronic proforma, information was extracted on URL, organisation, country of origin, focal health condition(s), target audience (consumer/carer/professional) and variables for quality rating (see below).

### Quality appraisal

Quality appraisal of each website was undertaken by one reviewer, with random checks performed on 10% by a second reviewer. The quality of websites was evaluated using a widely used tool for this purpose,^31^ the AMA benchmarks.^18^ AMA benchmarks are concerned with whether websites provide basic information on authorship (including credentials and affiliations), attribution (including references where applicable), currency (the dates content was added/reviewed) and disclosure of website ownership/sponsorship and potential conflicts of interest. We abandoned efforts to assess the quality of self-management content after tools designed for assessing information about health interventions were found to be a poor fit.^32,33^

Readability of written content was assessed using the most common approach^25^—the Flesch–Kincaid Grade Level Index.^34^ We followed previous recommendations that grade 8 should be considered the highest level acceptable for health information to be regarded as accessible by the general public.^19^

### Synthesis

Each website was classified according to the taxonomy shown in Table 3 by one reviewer, with random checks performed on 10% by a second reviewer. This taxonomy was developed for a Cochrane review by Howell *et al.*^12^ and includes self-management strategies found to be effective by previous research.^12^ To enable description of content related to breathlessness crises, this taxonomy was supplemented by recommendations for patient education on this topic provided by the ATS.^7^ Judgments regarding risks to safety were based on a review of related literature and the group’s clinical expertise.

## Figures and Tables

**Table 1:** Numbers of web pages searched via the Google search engine in June 2015

*Term*	*Search results*	
	*Results (millions)*	*Results hand-searched*
Breathless	19.5	10
Shortness of breath	10.6	77
Dyspnoea	3.4	40
Wheeze	0.72	30
Difficulty breathing	52	10

**Table 2:** Characteristics of 44 websites identified as providing support for self-management of chronic breathlessness

*Characteristic*	*Number (%)*
*Country*	
USA	28 (64)
UK	5 (11)
Canada	6 (14)
Australia	3 (7)
New Zealand	1 (2)
Europe	1 (2)
	
*Author*	
National Health Organisation	16 (36)
Other health	20 (45)
Health professional	6 (14)
User	3 (7)
	
*Format*	
Written Information	23 (52)
Video	16 (36)
Both	5 (11)
	
*Target audience*^a^	
People with breathlessness	45 (100)
Family/carers	8 (18)
Health professionals	4 (9)
	
*Health condition*^a^	
COPD	27 (61)
Cancer	9 (20)
Heart failure	3 (7)
Unspecified	15 (34)

Abbreviation: COPD, chronic obstructive pulmonary disease.

a Some websites included contents for more than one audience and health condition.

**Table 3:** Classification of breathlessness self-management interventions using evidence-based recommendations

*Types of breathlessness self-management programmes shown to be effective by research*^11^		*ATS guidance on support for dyspnoea crisis*^27^
Structured education	Information about breathlessness and its management including the actions patients can take: breathing and coughing techniques; energy conservation; health lifestyle behaviour change to increase activity/exercise; smoking cessation; management of roles and relationships; strategies for coping.	
		
Breathing techniques skill training	Inclusion of specific breathing techniques and opportunities for skill feedback and monitoring.	Breathing retraining including pursed lip breathing, slowed pattern of breathing, prolonged exhalation and posture modification.
		
Cognitive symptom management skill training	Coping training for symptoms, emotions and stress: therapeutic support; relaxation therapy.	Relaxation techniques, mindfulness meditation, guided imagery and distraction strategies (e.g., music, TV, reading by self or caregiver)
		
Goal setting and action planning	Using a short-term plan of action for exacerbations or panic breathing episodes and to reach goals.	Use of a written action plan that includes appropriate administration and dosing of medications and stepwise titration regimens
		
Problem-solving skills	Solution implementation and evaluation/monitoring of results; key messages and support for decision-making; skills of self-tailoring to facilitate adapting lifestyle to accommodate symptoms and to adopt health lifestyle change such as exercise: with or without surveillance; adherence measure.	
		
Coaching in use of support services	Awareness of resources and navigating skills to gain access.	
		
Partnership with healthcare providers	Ways to communicate effectively with providers.	
		
Enhancement of self-efficacy	Teaching for performance mastery, modelling, re-interpretation of symptoms and social persuasion (peer support/modelling).	
		
Other		• Basic facts about causes and triggers of dyspnoea crises. • How to identify signs and symptoms that are an indication of a dyspnoea crisis. • How to recognise and measure changes from baseline for both intensity of dyspnoea and an affective component (anxiety or distress). • Appropriate and individualised use of oxygen, ventilation and/or fans.
