# Supplementary material for: Content and quality of websites supporting self-management of chronic breathlessness in advanced illness: a systematic review
Source: NPJ Prim Care Respir Med. 2016 May 26;26:16025–. doi: 10.1038/npjpcrm.2016.25 (PMC4881311; doi:10.1038/npjpcrm.2016.25)
Supplement: Supplementary Table S1 [file npjpcrm201625-s1.doc]

**Supplementary Table 1.** Authorship, URLs, health conditions, target audience and content of 44 websites offering advice on self-management of chronic breathlessness

| **Website author** | **URL*** | **Health Condition** | **Target Audience** | **Structured education** | **Breathing and exercise techniques** | **Cognitive symptom management** | **Action planning** | **Problem solving** | **Use of support services** | **Partnership with HPs** | **Managing breathlessness crises** | **Monitoring breathlessness** | **Lifestyle factors** | **Environmental factors** | **Using treatments** | **End of Life issues** |
| --- | --- | --- | --- | --- | --- | --- | --- | --- | --- | --- | --- | --- | --- | --- | --- | --- |
| American Association for Respiratory Care | <http://www.yourlunghealth.org/healthy_living/articles/dance/> | NS | P | ✓ | ✓ |  |  |  | ✓ | ✓ |  |  | ✓ | ✓ | ✓ |  |
| American Cancer Society | <http://www.cancer.org/treatment/treatmentsandsideeffects/physicalsideeffects/dealingwithsymptomsathome/caring-for-the-patient-with-cancer-at-home-shortness-of-breath> | Cancer | P, HP |  | ✓ |  |  |  | ✓ |  | ✓ |  |  | ✓ |  |  |
| American College of Chest Physicians | <http://www.chestnet.org/Foundation/Patient-Education-Resources/COPD> | COPD | P | ✓ | ✓ | ✓ |  |  |  |  |  |  | ✓ | ✓ | ✓ |  |
| American Heart Association | <http://circ.ahajournals.org/content/108/2/e11.full> | COPD/  NS | P |  |  |  | ✓ |  |  |  |  |  | ✓ | ✓ |  |  |
| American Lung Association | <http://www.lung.org/> | COPD | P,HP |  |  |  | ✓ |  | ✓ |  |  |  | ✓ |  |  |  |
| American Thoracic Society | <http://www.thoracic.org/patients/patient-resources/fact-sheets-az.php> | NS | P, C | ✓ | ✓ | ✓ | ✓ |  | ✓ | ✓ | ✓ |  | ✓ | ✓ | ✓ |  |
| Asthma Foundation New Zealand | <http://asthmafoundation.org.nz/your-health/manage-your-copd/> | COPD | P |  | ✓ |  | ✓ |  | ✓ |  | ✓ |  | ✓ |  | ✓ |  |
| British Columbia Lung Foundation | <https://www.youtube.com/watch?v=wPg7h4N9Xnc> | COPD | P |  | ✓ |  |  |  |  |  |  |  |  | ✓ |  |  |
| British Lung Foundation | <http://www.blf.org.uk/Page/Breathlessness-lung-health> | COPD/  NS | P, C |  | ✓ |  | ✓ |  | ✓ | ✓ | ✓ |  | ✓ | ✓ | ✓ |  |
| Burke Rehabilitation and Research | <https://www.youtube.com/watch?v=VR7QnSnHmBU> | NS | P |  | ✓ |  |  |  |  |  |  |  |  |  |  |  |
| Canadian Lung Association | <https://www.lung.ca/lung-health/get-help> | COPD | P |  | ✓ |  | ✓ |  | ✓ | ✓ | ✓ |  | ✓ | ✓ | ✓ |  |
| Canadian Thoracic Society | <http://www.respiratoryguidelines.ca/guideline/chronic-obstructive-pulmonary-disease> | COPD | P, HP |  |  |  | ✓ |  |  |  |  |  |  |  |  |  |
| Cancer Research UK | <http://www.cancerresearchuk.org/about-cancer/coping-with-cancer/coping-physically/breathing/treatment/how-you-can-help-yourself> | Cancer | P |  | ✓ | ✓ | ✓ |  | ✓ |  | ✓ |  | ✓ |  |  |  |
| Central Grampians Palliative care | <https://www.youtube.com/watch?v=nHNu_tvDdqU> | NS | P |  | ✓ |  |  |  |  |  |  |  |  |  | ✓ |  |
| Chemocare | <http://chemocare.com/chemotherapy/side-effects/dyspnea.aspx> | Cancer | P |  | ✓ | ✓ |  |  | ✓ | ✓ | ✓ |  | ✓ | ✓ | ✓ |  |
| Chest, Heart and Stroke Scotland | <http://www.chss.org.uk/> | COPD/  heart failure | P, C |  | ✓ | ✓ | ✓ | ✓ | ✓ |  | ✓ | ✓ | ✓ | ✓ | ✓ |  |
| Cleveland Clinic | <http://my.clevelandclinic.org/health/diseases_conditions/hic_Understanding_COPD/hic_Pulmonary_Rehabilitation_Is_it_for_You/hic_Positions_to_Reduce_Shortness_of_Breath> | COPD | P |  | ✓ |  |  |  |  |  |  |  |  |  |  |  |
| COPD Foundation USA | <https://www.youtube.com/watch?v=W4kWLqvwzGM> | COPD | P |  | ✓ |  |  |  | ✓ |  |  |  | ✓ |  |  |  |
| COPDTV.com | <https://www.youtube.com/watch?v=RxPRng3FRD4> | COPD | P |  | ✓ |  |  |  |  |  |  |  |  |  |  |  |
| Ask Dr. Jo | <https://www.youtube.com/watch?v=-7-CAFhJn78> | COPD | P |  | ✓ |  |  |  |  |  |  |  |  |  |  |  |
| Dr. Nicholas Coleman | <https://www.youtube.com/watch?v=54vQxMFuj0I> | COPD | P |  |  |  |  |  | ✓ | ✓ |  |  | ✓ | ✓ | ✓ |  |
| FamilyDoctor.org | <http://familydoctor.org/familydoctor/en/health-tools/search-by-symptom/shortness-of-breath.html> | NS | P |  | ✓ |  | ✓ |  |  | ✓ | ✓ |  | ✓ |  |  |  |
| HamHealthSciences | <https://www.youtube.com/watch?v=KYj-T4Nd3h0> | NS | P |  | ✓ |  | ✓ |  |  |  |  |  |  |  |  |  |
| Healthline | <http://www.healthline.com/health/copd/living-with-copd> | COPD | P, C |  | ✓ | ✓ | ✓ |  | ✓ |  | ✓ |  | ✓ | ✓ | ✓ | ✓ |
| Heart Failure Association of the European Society of Cardiology | <http://www.heartfailurematters.org/en_GB/Warning-signs/Awakening-short-of-breath-needing-more-pillows> | Heart failure | P, C |  | ✓ |  |  |  | ✓ | ✓ | ✓ |  | ✓ | ✓ | ✓ | ✓ |
| Juransvinki Cancer Centre | <https://www.youtube.com/watch?v=nxw7rAmQeAI> | NS | P |  | ✓ |  |  |  |  |  |  |  |  |  |  |  |
| Lone Star College Students | <https://www.youtube.com/watch?v=iP1q8EM8IOw> | NS | P |  | ✓ |  |  |  |  |  |  |  |  |  |  |  |
| Lung Cancer Alliance USA | <http://www.lungcanceralliance.org/what-if-i-am-diagnosed/side-effect-management/shortness-of-breath-dyspnea.html> | Cancer | P |  | ✓ |  |  |  | ✓ |  | ✓ |  | ✓ | ✓ |  |  |
| Lung Foundation Australia | <http://lungfoundation.com.au/patient-area/self-management/> | Cancer | P, C | ✓ | ✓ | ✓ |  |  | ✓ | ✓ | ✓ |  | ✓ |  | ✓ | ✓ |
| Mayo Clinic | <http://www.mayoclinic.org/diseases-conditions/copd/basics/treatment/con-20032017> | COPD/  Cancer/NS | P |  | ✓ | ✓ | ✓ |  | ✓ |  | ✓ |  | ✓ |  | ✓ |  |
| MedicineNet.com | <http://www.medicinenet.com/script/main/art.asp?articlekey=3145> | COPD | P |  | ✓ |  |  |  | ✓ |  |  |  | ✓ | ✓ | ✓ |  |
| MedlinePlus | <https://www.nlm.nih.gov/medlineplus/ency/article/003075.htm> | COPD/  Cancer/  heart failure/  NS | P, C, HP |  | ✓ |  | ✓ |  | ✓ | ✓ | ✓ | ✓ | ✓ |  |  |  |
| Donna Wilson | <https://www.youtube.com/watch?v=fDUAIiOPXBY&list=PL1526604DDA1D82AD> | COPD/  Cancer | P |  | ✓ |  |  |  |  |  |  |  |  |  |  |  |
| Lois Pearl | <https://www.youtube.com/watch?v=DxJrLrqJ2nw> | NS | P |  | ✓ |  |  |  |  |  |  |  |  |  |  |  |
| National Health Service UK | <http://www.nhs.uk/conditions/shortness-of-breath/Pages/Introduction.aspx> | COPD/  NS | P |  | ✓ |  | ✓ |  | ✓ | ✓ | ✓ |  |  | ✓ | ✓ |  |
| National Jewish Health, USA | <http://www.nationaljewish.org/healthinfo/lifestyle/relax> | COPD | P |  | ✓ |  |  |  |  |  |  | ✓ |  |  | ✓ |  |
| OncoLink | <http://www.oncolink.org/coping/article.cfm?aid=1153&id=578&c=491> | Cancer | P |  | ✓ | ✓ |  |  | ✓ |  | ✓ |  | ✓ | ✓ | ✓ |  |
| Patient | <http://patient.info/health/chronic-obstructive-pulmonary-disease-leaflet> | COPD | P |  | ✓ |  |  |  | ✓ |  |  |  | ✓ | ✓ | ✓ |  |
| RealTimeHealth | <https://www.youtube.com/watch?v=UHTrlsqA89o> | COPD | P |  | ✓ |  |  |  |  |  |  |  | ✓ |  |  |  |
| The Credit Valley Hospital and Trillium Centre | <https://www.youtube.com/watch?v=HmTLdhDFXs0> | COPD | P |  | ✓ | ✓ |  |  | ✓ |  |  |  | ✓ | ✓ | ✓ |  |
| The New York Times | <https://www.youtube.com/watch?v=LideJlY9cAk> | COPD | P |  | ✓ |  |  |  |  |  |  |  | ✓ |  |  |  |
| University of Rochester Medical Centre | <http://www.urmc.rochester.edu/encyclopedia/content.aspx?ContentTypeID=34&ContentID=21274-1> | NS | P |  | ✓ | ✓ |  |  |  |  |  |  | ✓ |  |  |  |
| Visiting Nurse Service of New York | <https://www.youtube.com/watch?v=e6QXQ_g1ly0> | COPD | P, C |  |  |  |  |  |  |  |  |  | ✓ | ✓ | ✓ |  |
| WebMD | <http://www.webmd.com/lung/features/short-breath-tips-copd-breathlessness> | COPD | P |  | ✓ |  |  |  | ✓ |  |  |  |  |  |  |  |

* Where websites included more than one web-page, the URL is given for one web-page only. C = carers; COPD = chronic obstructive pulmonary disease; HP = health professionals; P = patients.
